# Supplementary material for: Analysis of PPARγ Signaling Activity in Psoriasis
Source: Int J Mol Sci. 2021 Aug 10;22(16):8603. doi: 10.3390/ijms22168603 (PMC8395241; doi:10.3390/ijms22168603)
Supplement: Supplementary file 1 [file ijms-22-08603-s001.zip › Supplemental materials_Analysis of PPARg signaling activity in psoriasis/Pathway models/Models images and html files/Interleukin-17 and interleukin-22 signaling in psoriasis/6602.html]

JUN


# Protein JUN

|  |  |
| --- | --- |
| URN | urn:agi-llid:3725 |
| Total Entities | 0 |
| Connectivity | 7144 |
| Name | JUN |
| Description | jun proto-oncogene |
| Notes | This gene is the putative transforming gene of avian sarcoma virus 17. It encodes a protein which is highly similar to the viral protein, and which interacts directly with specific target DNA sequences to regulate gene expression. This gene is intronless and is mapped to 1p32-p31, a chromosomal region involved in both translocations and deletions in human malignancies. [provided by RefSeq, Jul 2008] |
| Primary Cell Localization | Nucleus |
| Class | Transcription factor |

---

|  |  |
| --- | --- |
| Pathway | Osteoarthritis |
|  | Skin Aging |
|  | Oncogene Induced Cellular Senescence |
|  | MTHFR Activity Regulation |
|  | Melatonin Effects on Circadian Cycle |
|  | N-cell: Neurotensin Production |
|  | Dopamine Receptors Signaling |
|  | Prolactin Production |
|  | Thrombopoietin Receptors Signaling in Platelet Maturation |
|  | Cortisol in Resolving Inflammation |
|  | T-Cell Dependent B-Cell Activation |
|  | T-Cell Independent B-Cell Activation |
|  | Natural Killer Cell Activation through C-Type Lectin-like Receptors |
|  | Natural Killer Cell Activation through ITAM-Containing Receptors |
|  | Natural Killer Cell Activation through ITSM-Containing Receptors |
|  | Natural Killer Cell Activation |
|  | CD8+ T-Cell Activation |
|  | Plasmin Effects in Inflammation |
|  | Toll-like Receptor Independent Sterile Inflammation |
|  | Lipoxin A4/FPR2-Related Neutrophil Depression |
|  | Neutrophil Activation via LTB4/Polyisoprenyl Phosphate Signaling |
|  | Mast-Cell Activation without Degranulation through CXCR4 Signaling |
|  | CD16/CD14 Proinflammatory Monocyte Activation |
|  | anti-Inflammatory Function of Macrophage M2 Lineage |
|  | AXL Receptor Inhibits Macrophages and Dendritic Cells Function |
|  | Neutrophil Recruitment and Priming |
|  | Vascular Smooth Muscle Cell/Pericyte Migration and Proliferation |
|  | Vascular Endothelial Cell Activation by Blood Coagulation Factors |
|  | Adipokines Production by Adipocyte |
|  | Nociception Expression Targets Signaling |
|  | NTSR1 Expression Targets |
|  | ADRA1A -> IL6 Production |
|  | CNR1/2 -> IL1B/2/4/6/10 Production |
|  | DRD2 Expression Targets |
|  | DRD1/5 Expression Targets |
|  | CHRM1 -> IL2 Production |
|  | PTGER2/3 -> Inflammation-Related Expression Targets |
|  | IL1B Expression Targets -> Nociception |
|  | Melatonin in Cell Survival and Antioxidant Response |
|  | Cell Cycle Overiew |
|  | G0/G1 Phase Transition |
|  | SCF/FBXW7 Complex |
|  | Proteins Involved in Atherosclerosis |
|  | Dendritic Cells Function in Atherosclerosis |
|  | RAGE/AGER and S100 Proteins in Cardiovascular Injury |
|  | Proteins Involved in Hypertrophic Cardiomyopathy |
|  | Cardiomyocyte Hypertrophy |
|  | Hypertrophic Cardiomyopathy |
|  | Endothelin Signaling in Arterial Hypertension |
|  | Arterial Hypertension |
|  | Endothelial Cell Dysfunction in Arterial Hypertension |
|  | Aldosterone Synthesis Chronic Regulation |
|  | Smooth Muscle Cell Dysfunction in Arterial Hypertension |
|  | TRPM4/6/7/8 Signaling in Arterial Hypertension (Hypothesis) |
|  | Myocardial Remodeling |
|  | Proteins Involved in Myocardial Ischemia |
|  | Sebocyte Proliferation in Acne Vulgaris |
|  | Inflammatory Reaction in Acne Vulgaris |
|  | Mast-Cells Activation in Atopic Dermatitis |
|  | IL17 Signaling in Psoriasis |
|  | Proteins Involved in Psoriasis |
|  | IL22 Induces Keratinocyte Proliferation in Psoriasis |
|  | Proteins Involved in Photoaging |
|  | Th0-Cell Aberrant Activation in Systemic Lupus Erythematosus |
|  | B-Cell Activation in Systemic Lupus Erythematosus |
|  | Acute Cytotoxic CD8+T-Cell Response against Melanocytes |
|  | Humoral Immunity in Vitiligo |
|  | CD8+ T-Cell Response to Self-Determinants in Diabetes |
|  | Natural Killer T-Cell Roles in Diabetes Mellitus Type 1 |
|  | Natural Killer Cell in Diabetes Mellitus Type 1 |
|  | Peripheral Tolerance to Autoantigens Recession in Diabetes Mellitus Type 1 |
|  | Advanced Glycation End Products Pathway in Diabetic Microangiopathy |
|  | Protein Kinase C in Diabetic Microangiopathy |
|  | Peripheral Tissue Microangiopathy |
|  | Tubular Cell Dysfunction in Progressive Diabetic Nephropathy |
|  | Endothelial Cell Dysfunction in Progressive Diabetic Nephropathy |
|  | Endothelial Cell Dysfunction in Early Diabetic Nephropathy |
|  | Mesangial Cell Dysfunction in Diabetic Nephropathy |
|  | Microangiopathy in Diabetic Neuropathy |
|  | Proliferative Diabetic Retinopathy |
|  | Proteins Involved in Insulin Resistance |
|  | Adiponectin Synthesis Declines in Insulin Resistance |
|  | Hepatic Stellate Cells in non-Alcoholic Fatty Liver Disease |
|  | beta-Cell Destruction through Cytokines in Diabetes Mellitus |
|  | beta-Cell Mass Regulation |
|  | Hypothyrodism Associated Hyperprolactinemia |
|  | DRD2 Pharmacological Inhibition in Hyperprolactinemia |
|  | Ethanol Induced Efects in Hyperprolactinemia |
|  | Thyroid Dysfunction |
|  | GNRH Signaling Impairment in Hypogonadotrophic Hypogonadism |
|  | Myocyte Dysfunction in Hypoparathyroidism |
|  | Adipokines Production by Adipocyte Impaired in Obesity |
|  | Prolactinoma Overiew |
|  | CD8+ T-Cell Response in Celiac Disease |
|  | Proteins Involved in Colorectal Neoplasms |
|  | GUCY2C Signaling in Colorectal Cancer |
|  | OipA Signaling in Helicobacter Pylori Infection |
|  | CagA Phosphorylation Dependent Signaling |
|  | Peutz-Jeghers Syndrome |
|  | Epithelial Cell in the Innate Immune Response in Ulcerative Colitis |
|  | T-Cell Cytotoxic Response in Ulcerative Colitis |
|  | Proteins Involved in Acute Myeloid Leukemia |
|  | Antiphospholipid Antibodies in Endothelial Cell |
|  | Proteins Involved in Antiphospholipid Syndrome |
|  | B-Cell Acute Lymphoblastic Leukemia |
|  | B-Cell Chronic Lymphocytic Leukemia |
|  | Chronic Myeloid Leukemia |
|  | Follicular Lymphoma |
|  | Osteoclast Activation in Multiple Myeloma |
|  | Multiple Myeloma |
|  | Antiphospholipid Antibodies and Endothelial Cell Interaction |
|  | T-Cell Acute Lymphoblastic Leukemia |
|  | Proteins Involved in Hepatocellular Carcinoma |
|  | NOTCH Signaling in Hepatocellular Carcinoma |
|  | WNT Signaling in Hepatocellular Carcinoma |
|  | Hepatocellular Carcinoma |
|  | Growth Factor Signaling in Hepatocellular Carcinoma |
|  | Pancreatic Ductal Carcinoma |
|  | Growth Factor Signaling in Pancreatic Neoplasms |
|  | Pancreatic Neuroendocrine Tumors |
|  | TRPM7/8 in Pancreatic Adenocarcinoma |
|  | TGFBR Signaling in Pancreatic Neoplasms |
|  | TSLP Signaling in Bronchial Epithelial Cell in Asthma |
|  | Airway Smooth Muscle Cell High Rate Proliferation |
|  | Osteoclast Activation in Gout |
|  | Monocytes Function in Gout |
|  | TNF and IL1B Induce Metalloproteinase Synthesis in Osteoarthritis |
|  | Ovulation Block |
|  | FSH Action in Polycystic Ovary Syndrome |
|  | Synovial Fibroblast Activation in Psoriatic Arthritis |
|  | Keratinocyte Activation in Psoriatic Arthritis |
|  | Proteins Involved in Psoriatic Arthritis |
|  | PTPN22 Role in Rheumatoid Arthritis |
|  | Synovial Fibroblast Proliferation in Rheumatoid Arthritis |
|  | Osteoclast Activation in Rheumatoid Arthritis |
|  | Synovial Fibroblast Activation by Citokines in Rheumatoid Arthritis |
|  | TLR2 Induced Synovial Fibroblast Activation in Rheumatoid Arthritis |
|  | Autocrine Cytokine/Chemokine Loops in Systemic Scleroderma |
|  | B-Cells Function in Systemic Scleroderma |
|  | Skin Fibrosis |
|  | CCR5 Signaling in Macrophages |
|  | CD8+ T-Cell Action Impairment in HIV Type 1 Infection |
|  | Diabetes Induced Periodontitis |
|  | Estrogen Effects on Pregnancy Gingivitis |
|  | Majeed Syndrome |
|  | Hereditary Breast and Ovarian Cancer Syndrome |
|  | Cowden Syndrome |
|  | Proteins Involved in Huntington Disease |
|  | Oxytocin Signaling in Brain Nerve Cell (Hypothesis) |
|  | IL6 Promotes Inflammation in Muscular Dystrophies |
|  | IL6 Overexpression After Contraction in Skeletal Muscles in Muscular Dystrophies |
|  | Ca2+ Overload in Muscular Dystrophies |
|  | Muscular Dystrophies |
|  | Dystrophin Glycoprotein Complex Signaling in Muscular Dystrophies |
|  | IGF1 Role in Muscle Hypertrophy |
|  | Proteins Involved in Osteopetrosis |
|  | Osteoblast Function Decline in Osteoporosis |
|  | Osteoclast Activation in Postmenopause |
|  | Proteins Involved in Alzheimer's Disease |
|  | Metals and Amyloid beta Toxicity in Alzheimer's Disease |
|  | Apoptosis and Immediate Early Gene Activation in Epileptiform Disorders |
|  | Glioma Invasion Signaling |
|  | Proteins Involved in Medulloblastoma |
|  | Proteins Involved in Parkinson's Disease |
|  | Proteins Involved in Amyotrophic Lateral Sclerosis |
|  | Microglia and Motor Neuron Interaction Dysregulation |
|  | Proteins Involved in Breast Cancer Related to WNT Signaling Pathway |
|  | Proteins Involved in Breast Cancer Related to ESR1 Signaling Pathway |
|  | Proteins Involved in Breast Cancer Related to NOTCH Signaling Pathway |
|  | WNT Signaling in Breast Cancer |
|  | Proteins Involved in Breast Cancer Related to ERBB2/VEGFR/Akt Signaling Pathway |
|  | ERBB/VEGFR/Akt Signaling in Breast Cancer |
|  | IGF1R/AKT Signaling in Breast Cancer |
|  | ESR1/ERBB Positive Luminal Breast Cancer |
|  | Proteins Involved in Breast Cancer Related to IGF1R/Akt Signaling Pathway |
|  | Breast Cancer |
|  | ESR1 Signaling in Breast Cancer |
|  | NOTCH1 Signaling in Breast Cancer |
|  | Endometrial Cancer |
|  | Endometrioid Endometrial Cancer |
|  | Clear Cell Endometrial Cancer and Papillary Serous Endometrial Cancer |
|  | Angiogenesis in Endometriosis |
|  | Coagulation Factors and PAR1/2 Receptors (F2R/F2RL1) in Endometriosis |
|  | Leptin Role in Endometriosis |
|  | Proteins Involved in High-Grade Serous Ovarian Carcinoma |
|  | Endometrioid Ovarian Carcinoma |
|  | Proteins Involved in Endometrioid Ovarian Carcinoma |
|  | Clear Cell Ovarian Carcinoma |
|  | Proteins Involved in Mucinous Ovarian Carcinoma |
|  | High-Grade Serous Ovarian Carcinoma |
|  | Low-Grade Serous Ovarian Carcinoma |
|  | Proteins Involved in Low-Grade Serous Ovarian Carcinoma |
|  | Ovarian Cancer |
|  | Proteins Involved in Clear Cell Ovarian Carcinoma |
|  | Mucinous Ovarian Carcinoma |
|  | MIF Role in Cholesteatoma |
|  | HMGB1/RAGE Signaling in Middle Ear Cholesteatoma |
|  | Subepithelial Fibroblasts in Middle Ear Cholesteatoma |
|  | Cholesteatoma |
|  | HPV Infection and Cancer |
|  | HPV E5 Regulates EDNRA and EGFR Signaling Pathway in Keratinocytes |
|  | Kruppel-like Factor 6 Signaling in Prostate Cancer |
|  | TRPM8 Effects in Prostate Cancer (Hypothesis) |
|  | ER Stress in Macular Degeneration |
|  | Steroids Induced Cataract |
|  | VEGFA in Neovascular Glaucoma |
|  | PGF2-alpha Effect on Treatment |
|  | MUC5AC Expression by NTHi and S. Pneumoniae |
|  | Alveolar Macrophage Negative Regulation Declined in COPD |
|  | Alveolar Macrophages Dysfunction in COPD |
|  | VEGFA Low Expression Level in Chronic Bronchitis |
|  | T-Cell Cytotoxic Activation |
|  | Alveolar Epithelial Cell Dysfunction in COPD |
|  | WNT Signaling in Cystic Kidney Disease |
|  | Polycystins Mutation Effects in Cystic Kidney Disease |
|  | Plasmin Role in Glomerulonephritis |
|  | Endothelial Cell Dysfunction in Glomerulonephritis |
|  | Mesangial Cell Dysfunction in Glomerulonephritis |
|  | Interstitial Fibroblasts in Pyelonephritis |
|  | CD38/CD3 -> JUN/FOS/NF-kB Signaling in T-cell Proliferation |
|  | CD38/CD19 -> JUN/FOS/NF-kB Signaling in B-cell Proliferation |
|  | CD38 -> cADPR/Calcium Signaling |
|  | S100B Expression Targets |
|  | ANGPT2 -> STAT Expression Targets |
|  | ANGPT2 -> AP-1/CREBBP/MYC Expression Targets |
|  | AREG -> AP-1 Expression Targets |
|  | Noradrenaline/Gs Expression Targets |
|  | Dopamine/Gs Expression Targets |
|  | Noreadrenaline/Gq Expression Targets |
|  | Epinephrine/Gs Expression Targets |
|  | Acetylcholine Expression Targets |
|  | Serotonin/Gq Expression Targets |
|  | Epinephrine/Gq Expression Targets |
|  | Dopamine/Gi Expression Targets |
|  | B-Cell Receptor -> AP-1 Signaling |
|  | BMP2/BMPR2 Expression Targets |
|  | BMP4/BMPR2 Expression Targets |
|  | BMP7/BMPR2/ACVR2 Expression Targets |
|  | BTC -> AP-1/ATF/CREB Expression Targets |
|  | Elevated Receptors -> Expression Targets in Bone and Joint |
|  | AGTR1 -> ARRB1/ARRB2 Signaling |
|  | CD19 Expression Targets |
|  | CD81 -> AP-1 Expression Targets |
|  | CD247 Expression Targets |
|  | gamma Globulins Expression Targets |
|  | CD19/CR2 Expression Targets |
|  | CD72 -> CREB/CREBBP Expression Targets |
|  | CD8 -> ATF/CREB/CREBBP Expression Targets |
|  | CD80 -> NF-kB Expression Targets |
|  | CD86 -> NF-kB Expression Targets |
|  | Elevated Receptors -> Expression Targets in Myocardium |
|  | CXCL12 Expression Targets |
|  | CSF2 -> STAT Expression Targets |
|  | LIF Expression Targets |
|  | Clozapine Induced Granulocytopenia |
|  | EGF -> CTNN Expression Targets |
|  | EGF -> AP-1/ATF Expression Targets |
|  | EGF -> CREB/CREBBP/ELK/SRF/MYC Expression Targets |
|  | EGF -> MEF/MYOD/NFATC Expression Targets |
|  | EGF -> STAT Expression Targets |
|  | EGFR -> AP-1/ATF2 Signaling |
|  | EGFR -> AP-1/CREB/ELK/SRF/MYC Signaling |
|  | EREG -> AP-1/ATF Expression Targets |
|  | Erythropoietin -> NF-kB Expression Targets |
|  | Erythropoietin -> AP-1/MYC/CREB Expression Targets |
|  | Erythropoietin -> ELK/SRF Expression Targets |
|  | F2 -> AP-1/CREB/ELK/SRF/SP1 Expression Targets |
|  | F2 -> STAT1/NF-kB Expression Targets |
|  | PLG -> AP-1/CREB/ELK/SRF/SP1 Expression Targets |
|  | PLG -> STAT1/NF-kB Expression Targets |
|  | FGF9 -> AP-1/CREB/MYC Expression Targets |
|  | FGF10 -> AP-1/CREB/CREBBP/MYC Expression Targets |
|  | FGF2 -> STAT Expression Targets |
|  | FGF1 -> AP-1/CREB/ELK/SRF/MYC Expression Targets |
|  | FGF18 -> STAT Expression Targets |
|  | FGF18 -> AP-1/CREB Expression Targets |
|  | FGF1 -> STAT Expression Targets |
|  | FGF4 -> AP-1/MYC Expression Targets |
|  | FGF7 -> AP-1/CREB/CREBBP/MYC Expression Targets |
|  | FGF2 -> AP-1/CREB/CREBBP/ELK/SRF/MYC Expression Targets |
|  | FGFR -> AP-1/CREB/CREBBP/ELK/SRF/MYC Signaling |
|  | FrizzledR -> JUN/PAX2 Signaling |
|  | GCG Expression Targets |
|  | FSHR Expression Targets |
|  | VIP Expression Targets |
|  | GH1/GHR -> NF-kB/ELK/SRF/MYC Expression Targets |
|  | GH1/GHR -> STAT Expression Targets |
|  | GH1/PRLR Expression Targets |
|  | HBEGF -> AP-1/ATF Expression Targets |
|  | HGF -> STAT Expression Targets |
|  | HGF -> AP-1/CREB/ELK/SRF/MYC Expression Targets |
|  | HGFR -> AP-1/CREB/MYC Signaling |
|  | TAM Receptor Signaling Activation in Cancer |
|  | G0/G1 Cell Cycle Phase Transition Activation in Cancer |
|  | RAS/RAF/MAPK Signaling Activation in Cancer |
|  | WNT Canonical Signaling Activation in Cancer |
|  | Asbestosis- and Silicosis-Associated Inflammation Provokes Cancer Development |
|  | RAS/RAF/MAPK Signaling Activation by Blocking of Tumor Suppressors |
|  | WNT Signaling Activation by Blocking of Tumor Suppressors |
|  | TGFB Signaling activation by Blocking of Tumor Suppressors |
|  | VEGFA Dependent Angiogenesis in Cancer |
|  | ANGPT/TEK Stimulates Endothelial Cell Migration in Cancer |
|  | VEGF Independent Angiogenesis in Cancer |
|  | Integrins in Cancer Cell Motility, Invasion and Survival |
|  | MTOR/TORC in Cancer Cell Motility and Invasion |
|  | Hyaluronic Acid, CD44 and HMMR in Cancer Cell Invasion and Survival |
|  | Treg Cells Promote Immunosuppression in Cancer Immune Escape |
|  | IGF1 -> MEF/MYOD/MYOG Expression Targets |
|  | IGF1 -> STAT Expression Targets |
|  | IGF1 -> ELK/SRF/HIF1A/MYC/SREBF Expression Targets |
|  | INHBA/ACVR2/BMPR Expression Targets |
|  | Insulin -> STAT Expression Targets |
|  | Insulin -> CEBPA/CTNNB/FOXA/FOXO Expression Targets |
|  | Insulin -> MEF/MYOD Expression Targets |
|  | Insulin -> ELK/SRF/HIF1A/MYC/SREBF Expression Targets |
|  | CD19 -> JUN/ELK Signaling |
|  | ICAM1 -> AP-1/CREB/ELK/SRF Signaling |
|  | FibronectinR -> AP-1/ELK/SRF/SREBF Signaling |
|  | CTGF -> AP-1/CREB/MYC Expression Targets |
|  | ICAM1 Expression Targets |
|  | EGFR -> Expression Targets in Skin |
|  | IFNG/IFNR Expression Targets |
|  | IL7 Expression Targets |
|  | IL16 -> AP-1 Expression Targets |
|  | IL3 Expression Targets |
|  | IL6 Expression Targets |
|  | IL2 Expression Targets |
|  | PTPRJ Expression Targets |
|  | Leptin -> STAT Expression Targets |
|  | Leptin -> ELK/SRF Expression Targets |
|  | Prostaglandin F Expression Targets |
|  | Thromboxane A2 Expression Targets |
|  | LPA Expression Targets |
|  | PAF/Gq -> AP-1/ATF1/CREB/ERK/SRF Expression Targets |
|  | Dronabinol/Anandamide Expression Targets |
|  | Morphine Expression Targets |
|  | S1P Expression Targets |
|  | PGE1 Expression Targets |
|  | MTNR1 Signaling |
|  | NRG1 -> EP300/ETS/ETV/SP1 Expression Targets |
|  | NRG1 -> CREB/CREBBP/ELK/SRF/MYC Expression Targets |
|  | NRG1/Catenin Expression Targets |
|  | NRG1 -> STAT Expression Targets |
|  | NRG1 -> AP-1/ATF Expression Targets |
|  | Elevated Receptors -> Expression Targets in Nerve Tissue |
|  | NGF -> SMAD3/NF-kB Expression Targets |
|  | NGF -> AP-1/TP53/MYC Expression Targets |
|  | BDNF Expression Targets |
|  | NTF3 Expression Targets |
|  | NTF4 Expression Targets |
|  | Adenosine Expression Targets |
|  | PDGFB -> AP-1/CREB/MYC Expression Target |
|  | PDGFD -> AP-1 Expression Targets |
|  | PDGF -> AP-1/CREB/CREBBP/MYC Expression Targets |
|  | PDGF -> STAT Expression Targets |
|  | PDGFR -> AP-1/MYC Signaling |
|  | AVP/Gs -> CREB/ELK/SRF/AP-1/EGR Expression Targets |
|  | GNRH1 Expression Targets |
|  | NTS Expression Targets |
|  | GAST Expression Targets in GAST Expression Targets |
|  | GNRH2 Expression Targets |
|  | POMC Expression Targets |
|  | CCL11 Expression Targets |
|  | EDN1 Expression Targets |
|  | AGT -> CREB Expression Targets |
|  | NPY Expression Targets |
|  | AVP/Gq -> STAT Expression Targets |
|  | TAC1 Expression Targets |
|  | AVP/Gs -> STAT Expression Targets |
|  | EDN3 Expression Targets |
|  | AGT -> STAT Expression Targets |
|  | AVP/Gq -> CREB/ELK/SRF/AP-1/EGR Expression Targets |
|  | PRL/PRLR Expression Targets |
|  | PRL/GHR -> STAT Expression Targets |
|  | PRL/GHR -> NF/kB/ELK/SRF/MYC Expression Targets |
|  | PDCD1 -> AP-1 Expression Targets |
|  | AngiopoietinR -> AP-1 Signaling |
|  | EphrinB -> MAPK/JUN/FOS Signaling |
|  | NTRK -> AP-1/CREB/ELK/SRF/MYC/SMAD3/TP53 Signaling |
|  | CSF1 -> STAT Expression Targets |
|  | GAS6 -> AP-1/CREB Expression Targets |
|  | FIGF -> AP-1 Expression Target |
|  | KITLG -> STAT Expression Targets |
|  | KITLG -> AP-1/CREB/CREBBP/MYC Expression Targets |
|  | GDNF -> HSF1 Expression Targets |
|  | PAF Expression Targets |
|  | CSF1 -> AP-1/CREB/CREBBP/MYC Expression Targets |
|  | PGF -> AP-1/CREB/CREBBP/MYC Expression Targets |
|  | FLT3LG -> AP-1/CREB/CREBBP Expression Targets |
|  | TGFBR -> AP-1 Signaling |
|  | TGFBR1/AP-1 Signaling |
|  | MSTN/ACVR2/BMPR Expression Targets |
|  | TNF-alpha/TNFRSF1A Signaling |
|  | Lysophosphatidic Acid/LPARs Signaling |
|  | JNK/MAPK Signaling |
|  | Estrogens/ESR1 Genomic Canonical Signaling |
|  | MERTK Signaling |
|  | TGF-beta Signaling |
|  | BMPR2 Signaling |
|  | AXL Receptor Tyrosine Kinase Signaling |
|  | TNF-alpha/TNFRSF1B Signaling |
|  | VEGF Signaling |
|  | Estrogens/ESR1 non-Genomic Signaling |
|  | EGFR Signaling |
|  | WNT Planar Cell Polarity (PCP) non-Canonical Signaling |
|  | HRAS Signaling |
|  | ERK/MAPK Canonical Signaling |
|  | TCR -> AP-1 Expression Targets |
|  | TGFA -> STAT Expression Targets |
|  | TGFA -> CTNNB/CTNND Expression Targets |
|  | TGFA/AP-1/ATF Expression Targets |
|  | TGFA -> CREB/CREBBP/ELK-SRF/MYC Expression Targets |
|  | TGFB3-TGFBR2 Expression Targets |
|  | TGFB2-TGFBR2 Expression Targets |
|  | TGFB1-TGFBR2 Expression Targets |
|  | TGFB1-TGFBR1/AP-1 Expression Targets |
|  | TGFB1-ACVRL1 Expression Targets |
|  | FASLG Expression Targets |
|  | TNFSF10 Expression Targets |
|  | LTA Expression Targets |
|  | TNFSF14 Expression Targets |
|  | TNF -> AP-1 Expression Targets |
|  | CD72 -> AP-1 Expression Targets |
|  | TNFSF13 Expression Targets |
|  | TNFSF13B Expression Targets |
|  | EDA Expression Targets |
|  | EctodysplasinR -> AP-1 Signaling |
|  | TNFR -> AP-1/ATF/TP53 Signaling |
|  | NGFR -> AP-1/CEBPB/CREB/ELK/SRF/TP53 Signaling |
|  | TNFRSF1A -> AP-1/ATF/TP53 Signaling |
|  | Thrombopoietin -> AP-1/CREB/CREBBP/MYC Expression Targets |
|  | TLR4 -> AP-1 Expression Targets |
|  | TLR5 Expression Targets |
|  | TLR7 Expression Targets |
|  | IL1B -> NO Expression Targets |
|  | TLR4 -> AP-1/EGR1/HIF1A Expression Targets |
|  | IL1B -> PGE2 Expression Targets |
|  | TLR1 -> 2/6 Expression Targets |
|  | IL1B Expression Targets |
|  | IL1A Expression Targets |
|  | TLR9 Expression Targets |
|  | TLR -> AP-1 Signaling |
|  | VEGFA -> ATF/CREB/ELK-SRF Expression Targets |
|  | VEGFA -> STAT Expression Targets |
|  | VEGFA -> AP-1/CREBBP/MYC Expression Targets |
|  | VEGFA -> CTNNB/CTNND Expression Targets |
|  | VEGFR -> AP-1/CREB/MYC Signaling |
|  | WNT1 Expression Targets |
|  | WNT3A Expression Targets |
|  | WNT4 Expression Targets |
|  | WNT5A Expression Targets |
|  | WNT7A Expression Targets |
|  | pathway 3 |
|  | n |
|  | ace2 |
|  | prarg negative regulators, ps-positive |
|  | PPARG negative regulators |
|  | pparg expr target\_regulators |
|  | Neighbors of disease exacerbation |
|  | Neighbors of tumor progression |
|  | Neighbors of cancer progression |
|  | Neighbors of inflammatory status |
|  | Neighbors of overall survival |
|  | Neighbors of tumor size |
|  | Neighbors of mortality |
|  | Neighbors of survival rate |
|  | Neighbors of infarct size |
|  | Neighbors of survival time |
|  | Neighbors of functional recovery |
|  | Neighbors of insulin sensitivity |
|  | Neighbors of bacterial load |
|  | Neighbors of cancer survival |
|  | Neighbors of lethality |
|  | Neighbors of recurrence-free survival |
|  | Neighbors of macrophage count |
|  | Neighbors of long-term survival |
|  | Neighbors of treatment outcome |
|  | Neighbors of body weight |
|  | Neighbors of tumor promotion |
|  | Neighbors of morbidity |
|  | Neighbors of mortality rate |
|  | Neighbors of exhaustion |
|  | Neighbors of radioresistance |
|  | Neighbors of cell density |
|  | Neighbors of apoptosis rate |
|  | Neighbors of viral load |
|  | Neighbors of tumor grade |
|  | Neighbors of tumor resistance |
|  | Neighbors of renal hypertrophy |
|  | Neighbors of radiosensitivity |
|  | Neighbors of pregnancy outcome |
|  | Neighbors of metabolic response |
|  | Neighbors of disease exacerbation |
|  | Neighbors of tumor progression |
|  | Neighbors of cancer progression |
|  | Neighbors of inflammatory status |
|  | Neighbors of overall survival |
|  | Neighbors of tumor size |
|  | Neighbors of mortality |
|  | Neighbors of survival rate |
|  | Neighbors of infarct size |
|  | Neighbors of survival time |
|  | Neighbors of functional recovery |
|  | Neighbors of insulin sensitivity |
|  | Neighbors of bacterial load |
|  | Neighbors of cancer survival |
|  | Neighbors of lethality |
|  | Neighbors of recurrence-free survival |
|  | Neighbors of macrophage count |
|  | Neighbors of long-term survival |
|  | Neighbors of treatment outcome |
|  | Neighbors of body weight |
|  | Neighbors of tumor promotion |
|  | Neighbors of morbidity |
|  | Neighbors of mortality rate |
|  | Neighbors of exhaustion |
|  | Neighbors of radioresistance |
|  | Neighbors of cell density |
|  | Neighbors of apoptosis rate |
|  | Neighbors of viral load |
|  | Neighbors of tumor grade |
|  | Neighbors of tumor resistance |
|  | Neighbors of renal hypertrophy |
|  | Neighbors of radiosensitivity |
|  | Neighbors of pregnancy outcome |
|  | Neighbors of metabolic response |
|  | Neighbors of cell development |
|  | Neighbors of immune response |
|  | Neighbors of cell population |
|  | Neighbors of T-cell development |
|  | Neighbors of inflammatory response |
|  | Neighbors of wound healing |
|  | Neighbors of angiogenesis |
|  | Neighbors of innate immune response |
|  | Neighbors of cell function |
|  | Neighbors of macrophage activation |
|  | Neighbors of cell formation |
|  | Neighbors of osteoclast development |
|  | Neighbors of epithelial to mesenchymal transition |
|  | Neighbors of immunity |
|  | Neighbors of cell proliferative response |
|  | Neighbors of dendritic cell differentiation |
|  | Neighbors of cell survival |
|  | Neighbors of osteoclast differentiation |
|  | Neighbors of cell phenotype |
|  | Neighbors of T-cell proliferation |
|  | Neighbors of tissue remodeling |
|  | Neighbors of cell adhesion |
|  | Neighbors of T-cell activation |
|  | Neighbors of endothelial cell proliferation |
|  | Neighbors of fibrogenesis |
|  | Neighbors of tumor growth |
|  | Neighbors of dendritic cell development |
|  | Neighbors of tissue repair |
|  | Neighbors of cell count |
|  | Neighbors of cell infiltration |
|  | Neighbors of cell interaction |
|  | Neighbors of SMC proliferation |
|  | Neighbors of lumen formation |
|  | Neighbors of endothelial cell migration |
|  | Neighbors of adipogenesis |
|  | Neighbors of lipid storage |
|  | Neighbors of cell damage |
|  | Neighbors of pregnancy |
|  | Neighbors of monocyte differentiation |
|  | Neighbors of aging |
|  | Neighbors of cell motility |
|  | Neighbors of bone resorption |
|  | Neighbors of fibroblast proliferation |
|  | Neighbors of adipocyte differentiation |
|  | Neighbors of microglial activation |
|  | Neighbors of regeneration |
|  | Neighbors of cell migration |
|  | Neighbors of cancer growth |
|  | Neighbors of stem cell proliferation |
|  | Neighbors of smooth muscle cell migration |
|  | Neighbors of hepatic regeneration |
|  | Neighbors of transendothelial migration |
|  | Neighbors of osteoclast formation |
|  | Neighbors of cell invasion |
|  | Neighbors of heart function |
|  | Neighbors of hemopoiesis |
|  | Neighbors of bone remodeling |
|  | Neighbors of ossification |
|  | Neighbors of T-cell function |
|  | Neighbors of immune system function |
|  | Neighbors of lung development |
|  | Neighbors of autophagy |
|  | Neighbors of monocyte adhesion |
|  | Neighbors of transcription activation |
|  | Neighbors of senescence |
|  | Neighbors of hepatocyte proliferation |
|  | Neighbors of cell transdifferentiation |
|  | Neighbors of neuroprotection |
|  | Neighbors of stem cell differentiation |
|  | Neighbors of endothelial cell function |
|  | Neighbors of neuronal death |
|  | Neighbors of cell growth |
|  | Neighbors of ROS generation |
|  | Neighbors of epithelial cell proliferation |
|  | Neighbors of cancer cell growth |
|  | Neighbors of sensitization |
|  | Neighbors of macrophage function |
|  | Neighbors of cellular immune response |
|  | Neighbors of cellular senescence |
|  | Neighbors of colony formation |
|  | Neighbors of macrophage differentiation |
|  | Neighbors of cell differentiation |
|  | Neighbors of keratinocyte proliferation |
|  | Neighbors of stem cell migration |
|  | Neighbors of life span |
|  | Neighbors of psoriasis |
|  | Neighbors of inflammatory disease |
|  | Neighbors of rheumatoid arthritis |
|  | Neighbors of atherosclerosis |
|  | Neighbors of fibrosis |
|  | Neighbors of arthritis |
|  | Neighbors of injury |
|  | Neighbors of atherogenesis |
|  | Neighbors of vascular remodeling |
|  | Neighbors of metastasis |
|  | Neighbors of carcinogenesis |
|  | Neighbors of inflammation |
|  | Neighbors of tumor microenvironment |
|  | Neighbors of pathological angiogenesis |
|  | Neighbors of obesity |
|  | Neighbors of vascular disease |
|  | Neighbors of diabetes mellitus |
|  | Neighbors of asthma |
|  | Neighbors of pneumonia |
|  | Neighbors of hyperplasia |
|  | Neighbors of osteoarthritis |
|  | Neighbors of chronic obstructive pulmonary disease |
|  | Neighbors of reperfusion injury |
|  | Neighbors of airway inflammation |
|  | Neighbors of hepatitis |
|  | Neighbors of insulin resistance |
|  | Neighbors of infection |
|  | Neighbors of vascular injury |
|  | Neighbors of liver injury |
|  | Neighbors of neuroinflammation |
|  | Neighbors of sepsis |
|  | Neighbors of death |
|  | Neighbors of type 1 diabetes |
|  | Neighbors of autoimmune disease |
|  | Neighbors of ischemic stroke |
|  | Neighbors of dermatitis |
|  | Neighbors of neutrophil infiltration |
|  | Neighbors of heart disease |
|  | Neighbors of diabetic nephropathy |
|  | Neighbors of hepatocellular carcinoma |
|  | Neighbors of gastric cancer |
|  | Neighbors of colorectal cancer |
|  | Neighbors of cardiac remodeling |
|  | Neighbors of breast cancer |
|  | Neighbors of ischemia |
|  | Neighbors of systemic lupus erythematosus |
|  | Neighbors of melanoma |
|  | Neighbors of cartilage degeneration |
|  | Neighbors of interstitial fibrosis |
|  | Neighbors of neoplasm invasion |
|  | Neighbors of heart failure |
|  | Neighbors of lung disease |
|  | Neighbors of pulmonary fibrosis |
|  | Neighbors of cardiovascular disease |
|  | Neighbors of cancer |
|  | Neighbors of cerebral ischemia |
|  | Neighbors of neoplasm |
|  | Neighbors of neointima |
|  | Neighbors of atrophy |
|  | Neighbors of respiratory hypersensitivity |
|  | Neighbors of myocardial fibrosis |
|  | Neighbors of liver disease |
|  | Neighbors of prostate cancer |
|  | Neighbors of hypertrophy |
|  | Neighbors of ovarian cancer |
|  | Neighbors of pancreatic cancer |
|  | Neighbors of hypertension |
|  | Neighbors of intimal hyperplasia |
|  | Neighbors of kidney mesangial cell |
|  | Neighbors of osteoclast |
|  | Neighbors of T-cell |
|  | Neighbors of lung |
|  | Neighbors of liver |
|  | Neighbors of skin |
|  | Neighbors of brain |
|  | Neighbors of nerve |
|  | Neighbors of NF-kB family |
|  | Neighbors of JNK |
|  | Neighbors of IL1 family |
|  | Neighbors of PPAR |
|  | Neighbors of Jun/Fos |
|  | Neighbors of NFKBI |
|  | Neighbors of PKC |
|  | Neighbors of PI3K |
|  | Neighbors of STAT family |
|  | Neighbors of mitogen-activated protein kinase |
|  | Neighbors of cytokine |
|  | Neighbors of interleukin |
|  | Neighbors of JAK |
|  | Neighbors of PDGF |
|  | Neighbors of Ras GTPase |
|  | Neighbors of ERK1/2 |
|  | Neighbors of growth factor |
|  | Neighbors of PKA |
|  | Neighbors of LDL |
|  | Neighbors of NF-AT family |
|  | Neighbors of beta adrenoceptor |
|  | Neighbors of chorionic gonadotropin |
|  | Neighbors of IKBK |
|  | Neighbors of protein tyrosine kinase |
|  | Neighbors of NAD(P)H oxidase |
|  | Neighbors of SMAD subfamily |
|  | Neighbors of LXR |
|  | Neighbors of p38 |
|  | Neighbors of estrogen receptor |
|  | Neighbors of T-cell receptor |
|  | Neighbors of histone deacetylase |
|  | Neighbors of PDGF-BB |
|  | Neighbors of HIF-1 |
|  | Neighbors of GPCR |
|  | Neighbors of prostaglandin-endoperoxide synthase |
|  | Neighbors of aldehyde reductase |
|  | Neighbors of mitogen-activated protein kinase kinase |
|  | Neighbors of TSH |
|  | Neighbors of endothelin |
|  | Neighbors of proteasome endopeptidase complex |
|  | Neighbors of histone H3 |
|  | Neighbors of alpha2 adrenoceptor |
|  | Neighbors of vitronectin receptor (integrin) |
|  | Neighbors of PP2A |
|  | Neighbors of SAPK |
|  | Neighbors of WNT |
|  | Neighbors of gonadotropins |
|  | Neighbors of immunoglobulin |
|  | Neighbors of PP2B |
|  | Neighbors of phospholipase |
|  | Neighbors of endostatins |
|  | Neighbors of FSH |
|  | Neighbors of TORC1 |
|  | Neighbors of RA receptor |
|  | Neighbors of histone |
|  | Neighbors of Rho kinase |
|  | Neighbors of PDGF receptor |
|  | Neighbors of histone acetyltransferase |
|  | Neighbors of adrenoceptor |
|  | Neighbors of phosphodiesterase |
|  | sars-cov no binding |
|  | Figure 3\_Mixed network of dexamethasone targets |
|  | Figure 3\_Mixed network of dexamethasone targets |
|  | 1\_Inflammation in keratinocytes in the pathology of acne |
|  | 2\_Hyperseborrhea in acne vulgaris |
|  | 2\_Acute phase initiation in atopic dermatitis |
|  | 2\_Interleukin-17 and interleukin-22 signaling in psoriasis |
|  | 1\_TNF and IL1B provoke ECM degradation in osteoarthritis |
|  | 1\_Osteoclast-mediated bone resorption in postmenopausal women |
|  | 2\_Osteoblast-mediated bone demineralization |
|  | 1\_Osteoclast functional impairment in osteopetrosis |
|  | 1\_Formation of cysts in polycystic kidney disease |
|  | 3\_The role of interstitial fibroblasts in pyelonephritis |
|  | 3\_Mesangial cell dysfunction in glomerulonephritis |
|  | 1\_Local estrogen production stimulates ectopic endometrium proliferation |
|  | 3\_Enhanced angiogenesis in endometriosis |
|  | 1\_Disruption of luteinizing hormone and follicle-stimulating hormone secretion in PCOS |
|  | 3\_Endoplasmic reticulum stress in age-related macular degeneration |
|  | 2\_Pathogens stimulate mucins expression in the middle ear |
|  | 2\_Cardiomyocyte hypertrophy |
|  | 3\_Dysfunction of smooth muscle cells in hypertension |
|  | 1\_Cigarette smoke causes dysfunction and production of inflammatory mediators in alveolar epithelial cells |
|  | 3\_Role of alveolar macrophages in pulmonary tissue destruction in COPD |
|  | 3\_1\_Mucus accumulation in asthma |
|  | 4\_2\_Airway smooth muscle cells proliferation and airway tissue remodeling |
|  | 4\_Mucin hyperproduction in goblet and mucous cells in COPD |
|  | 2\_Intestine epithelial cells drive immunological responses in ulcerative colitis |
|  | 2\_CD8+ T-cell response and mucosal damage in celiac disease |
|  | 6\_p12I Viral protein promotes T-cell activation in HTLV-1 infection |
|  | 3\_Human papilloma virus E4 and E5 proteins in viral reproduction |
|  | 3\_1\_Beta-cell proliferation and compensation: proliferation |
|  | 3\_Islet beta-cell destruction in T1D |
|  | 1\_Microangiopathy in diabetic neuropathy |
|  | drug-target pairs binding |
|  | mapk1 |
|  | New Pathway |
|  | Interleukin-17 and interleukin-22 signaling in psoriasis |

---

|  |  |
| --- | --- |
| Group | Proteins Involved in Hereditary Breast and Ovarian Cancer Syndrome |
|  | Proteins Involved in Cowden Syndrome |
|  | other partners for SARS |

---

|  |  |
| --- | --- |
| Source | curated |
|  | ResNet |
|  | GO component |

---

|  |  |
| --- | --- |
| MedScan ID | 3725 |

---

|  |  |
| --- | --- |
| LocusLink ID | 3725 |
|  | 16476 |
|  | 24516 |
|  | 116476 |
|  | 298248 |
|  | 335916 |
|  | 280831 |

---

|  |  |
| --- | --- |
| Alias | LOC100288387 |
|  | AH119 |
|  | LOC100293034 |
|  | v-jun sarcoma virus 17 oncogene homolog (avian) |
|  | c-JUN |
|  | Jun |
|  | transcription factor c-Jun |
|  | LOC100291417 |
|  | Proto-oncogene C-JUN |
|  | v-jun avian sarcoma virus 17 oncogene homolog |
|  | OTTMUSP00000008346 |
|  | proto oncogene proteins c jun |
|  | Avian sarcoma virus 17 (v-jun) oncogene homolog |
|  | Jun proto-oncogene, AP-1 transcription factor subunit |
|  | jun A |
|  | Jun oncogene |
|  | RP23-135A21.5 |
|  | AP1\_HUMAN |
|  | v-jun sarcoma virus 17 oncogene homolog |
|  | jun proto-oncogene |
|  | Junc |
|  | OTTHUMP00000010036 |
|  | AP1 |
|  | p39 |
|  | AP-1 |
|  | Jun activation domain binding protein |
|  | activator protein 1 |
|  | enhancer-binding protein AP1 |
|  | transcription factor AP-1 |
|  | immediate early |
|  | Rjg-9 |
|  | Transcription factor AP I |
|  | AP I |
|  | activating protein I |
|  | activating protein 1 |
|  | immediate early gene |
|  | Ca125 |
|  | v-jun |
|  | c-jun-Ub |
|  | similar to v-jun sarcoma virus 17 oncogene homolog; Avian sarcoma virus 17 (v-jun) oncogene homolog |
|  | DKEY-86E18.3 |
|  | c-Jun protein |
|  | chromosome |
|  | fj36h07 |
|  | scaffold |
|  | wu:fj36h07 |
|  | zgc:65863 |

---

|  |  |
| --- | --- |
| GO ID | 0003677 |
|  | 0001228 |
|  | 0003700 |
|  | 0000981 |
|  | 0005096 |
|  | 0071837 |
|  | 0070412 |
|  | 0003723 |
|  | 0001102 |
|  | 0000980 |
|  | 0000978 |
|  | 0035497 |
|  | 0003682 |
|  | 0019899 |
|  | 0046982 |
|  | 0042803 |
|  | 0003713 |
|  | 0008134 |
|  | 0044212 |
|  | 0000976 |
|  | 0031625 |
|  | 0044389 |
|  | 0038095 |
|  | 0007265 |
|  | 0060395 |
|  | 0007568 |
|  | 0001525 |
|  | 0031103 |
|  | 0071276 |
|  | 0071277 |
|  | 0032870 |
|  | 0051365 |
|  | 0034614 |
|  | 0007623 |
|  | 0061029 |
|  | 0035026 |
|  | 0007612 |
|  | 0001889 |
|  | 0051899 |
|  | 0001774 |
|  | 0030224 |
|  | 0043922 |
|  | 0043392 |
|  | 0008285 |
|  | 0043524 |
|  | 0031953 |
|  | 0000122 |
|  | 1990441 |
|  | 0045892 |
|  | 0003151 |
|  | 0043923 |
|  | 0045740 |
|  | 2000144 |
|  | 0070374 |
|  | 0043547 |
|  | 0043065 |
|  | 0045597 |
|  | 0001938 |
|  | 0010634 |
|  | 0048146 |
|  | 0045657 |
|  | 0043525 |
|  | 1902895 |
|  | 0045944 |
|  | 0045893 |
|  | 1904707 |
|  | 0051090 |
|  | 0051726 |
|  | 0001836 |
|  | 0051591 |
|  | 0034097 |
|  | 0042493 |
|  | 0042542 |
|  | 0032496 |
|  | 0009612 |
|  | 0035994 |
|  | 0010033 |
|  | 0009314 |
|  | 0006366 |
|  | 0007179 |
|  | 0005829 |
|  | 0000790 |
|  | 0000228 |
|  | 0005719 |
|  | 0005654 |
|  | 0005634 |
|  | 0035976 |
|  | 0005667 |
|  | 0017053 |
|  | 0033613 |
|  | 0003690 |
|  | 0043565 |
|  | 0009987 |
|  | 0043066 |
|  | 0008284 |
|  | 0048661 |
|  | 0006355 |
|  | 0006351 |
|  | 0071222 |
|  | 0006955 |
|  | 0051091 |
|  | 0014070 |
|  | 0044822 |
|  | 0000982 |
|  | 0003705 |
|  | 0001077 |
|  | 0001190 |
|  | 0007184 |
|  | 0010941 |
|  | 0042127 |
|  | 0005100 |
|  | 0002755 |
|  | 0002756 |
|  | 0035666 |
|  | 0045087 |
|  | 0032321 |
|  | 0051403 |
|  | 0034166 |
|  | 0034134 |
|  | 0034138 |
|  | 0034142 |
|  | 0034146 |
|  | 0034162 |
|  | 0038123 |
|  | 0038124 |
|  | 0002224 |
|  | 0008063 |
|  | 0034130 |
|  | 0003702 |
|  | 0010843 |
|  | 0016563 |
|  | 0016564 |
|  | 0010552 |
|  | 0006350 |
|  | 0046983 |
|  | 0045941 |
|  | 0046332 |
|  | 0005515 |
|  | 0045449 |
|  | 0005622 |
|  | 0000074 |
|  | 0050875 |
|  | 0008270 |
|  | 0008151 |
|  | 0046686 |
|  | 0051597 |

---

|  |  |
| --- | --- |
| KEGG ID | hsa:3725 |
|  | mmu:16476 |
|  | rno:24516 |

---

|  |  |
| --- | --- |
| MedLine Reference | 11818961 |
|  | 12050152 |
|  | 12791271 |
|  | 12798298 |
|  | 12943993 |
|  | 10428834 |
|  | 10918580 |
|  | 10973489 |
|  | 2825349 |
|  | 9443941 |
|  | 11001927 |
|  | 11597910 |
|  | 11891228 |
|  | 12064606 |
|  | 12145210 |
|  | 12441106 |
|  | 12477932 |
|  | 12743595 |
|  | 12753154 |
|  | 12869527 |
|  | 1310930 |
|  | 14512277 |
|  | 14643899 |
|  | 15314684 |
|  | 15557322 |
|  | 15737994 |
|  | 15797868 |
|  | 15911351 |
|  | 16006965 |
|  | 16087680 |
|  | 16158334 |
|  | 17111371 |
|  | 2113275 |
|  | 2507134 |
|  | 7560090 |
|  | 7721748 |
|  | 8889548 |
|  | 9826657 |
|  | 10022836 |
|  | 10076871 |
|  | 10080190 |
|  | 10087299 |
|  | 10230788 |
|  | 10341238 |
|  | 10349636 |
|  | 10352021 |
|  | 10551403 |
|  | 10728913 |
|  | 10888866 |
|  | 10960769 |
|  | 11042159 |
|  | 11076861 |
|  | 11157052 |
|  | 11217851 |
|  | 11438667 |
|  | 11731234 |
|  | 11779499 |
|  | 11813303 |
|  | 11840290 |
|  | 11867524 |
|  | 11884386 |
|  | 11909859 |
|  | 11909946 |
|  | 11927562 |
|  | 11948401 |
|  | 12034730 |
|  | 12036911 |
|  | 12052899 |
|  | 12119282 |
|  | 12163468 |
|  | 12181357 |
|  | 12215258 |
|  | 12376107 |
|  | 12414794 |
|  | 12441295 |
|  | 12466851 |
|  | 12489182 |
|  | 12532319 |
|  | 12553907 |
|  | 12719996 |
|  | 12756228 |
|  | 12791272 |
|  | 1281302 |
|  | 12815619 |
|  | 12859204 |
|  | 12888549 |
|  | 12929935 |
|  | 12941696 |
|  | 1315307 |
|  | 1351769 |
|  | 1383034 |
|  | 14517347 |
|  | 1459456 |
|  | 14597166 |
|  | 14638681 |
|  | 14638694 |
|  | 14670820 |
|  | 14688262 |
|  | 14757751 |
|  | 14765123 |
|  | 15121835 |
|  | 15135887 |
|  | 15219855 |
|  | 15233917 |
|  | 15256779 |
|  | 15291758 |
|  | 15292661 |
|  | 15302867 |
|  | 15336319 |
|  | 15358865 |
|  | 15375216 |
|  | 15456874 |
|  | 15494506 |
|  | 15522312 |
|  | 15528206 |
|  | 15534203 |
|  | 15541355 |
|  | 15558021 |
|  | 15572149 |
|  | 15572151 |
|  | 15596805 |
|  | 15618518 |
|  | 15626733 |
|  | 15686869 |
|  | 15688012 |
|  | 15708845 |
|  | 15733037 |
|  | 15791649 |
|  | 15795322 |
|  | 15799023 |
|  | 15831482 |
|  | 15840658 |
|  | 15867431 |
|  | 15944151 |
|  | 15944271 |
|  | 15944387 |
|  | 15950217 |
|  | 15961692 |
|  | 16002216 |
|  | 16007074 |
|  | 16049073 |
|  | 1605850 |
|  | 16085472 |
|  | 16123044 |
|  | 16141072 |
|  | 16141073 |
|  | 16148154 |
|  | 16157693 |
|  | 16158333 |
|  | 16163348 |
|  | 16217033 |
|  | 16228289 |
|  | 1631061 |
|  | 16313514 |
|  | 16373341 |
|  | 16373364 |
|  | 16410637 |
|  | 16416088 |
|  | 16473943 |
|  | 16581799 |
|  | 16759155 |
|  | 16785994 |
|  | 16837500 |
|  | 16847049 |
|  | 1684952 |
|  | 1685142 |
|  | 16912279 |
|  | 16973441 |
|  | 17041602 |
|  | 17066317 |
|  | 17074809 |
|  | 17088247 |
|  | 17092936 |
|  | 17108173 |
|  | 1765007 |
|  | 1793595 |
|  | 1887331 |
|  | 1978328 |
|  | 2104845 |
|  | 2108401 |
|  | 2140597 |
|  | 2457172 |
|  | 2574141 |
|  | 2842750 |
|  | 3127828 |
|  | 3136397 |
|  | 3142691 |
|  | 3186736 |
|  | 3422745 |
|  | 7476995 |
|  | 7579389 |
|  | 7601910 |
|  | 7656403 |
|  | 7700631 |
|  | 7705639 |
|  | 7719022 |
|  | 7744871 |
|  | 7748560 |
|  | 7798322 |
|  | 7841657 |
|  | 7862133 |
|  | 7889400 |
|  | 7923577 |
|  | 7929053 |
|  | 7949731 |
|  | 7955078 |
|  | 7989745 |
|  | 8012112 |
|  | 8082881 |
|  | 8088797 |
|  | 8098006 |
|  | 8119710 |
|  | 8123597 |
|  | 8137749 |
|  | 8144618 |
|  | 8150544 |
|  | 8183239 |
|  | 8197114 |
|  | 8208542 |
|  | 8208545 |
|  | 8286110 |
|  | 8308027 |
|  | 8313375 |
|  | 8330736 |
|  | 8344255 |
|  | 8363569 |
|  | 8364887 |
|  | 8369223 |
|  | 8371760 |
|  | 8397366 |
|  | 8415706 |
|  | 8453995 |
|  | 8458421 |
|  | 8479285 |
|  | 8541477 |
|  | 8565820 |
|  | 8637721 |
|  | 8747598 |
|  | 8754282 |
|  | 8777432 |
|  | 8790912 |
|  | 8793856 |
|  | 8798505 |
|  | 8940147 |
|  | 9023306 |
|  | 9047395 |
|  | 9098922 |
|  | 9108083 |
|  | 9108383 |
|  | 9128250 |
|  | 9141200 |
|  | 9154819 |
|  | 9169087 |
|  | 9344659 |
|  | 9356505 |
|  | 9405446 |
|  | 9459310 |
|  | 9524251 |
|  | 9570962 |
|  | 9598328 |
|  | 9858769 |
|  | 9876179 |
|  | 9878062 |
|  | 10026157 |
|  | 10188588 |
|  | 10196196 |
|  | 10220381 |
|  | 10330159 |
|  | 10357819 |
|  | 10421840 |
|  | 10478848 |
|  | 10488148 |
|  | 10490649 |
|  | 10497212 |
|  | 10506225 |
|  | 10509564 |
|  | 10523647 |
|  | 10637231 |
|  | 10644332 |
|  | 10799874 |
|  | 10878360 |
|  | 11022789 |
|  | 11053448 |
|  | 11254713 |
|  | 11260070 |
|  | 11274169 |
|  | 11278277 |
|  | 11278640 |
|  | 11278891 |
|  | 11316804 |
|  | 11331585 |
|  | 11371641 |
|  | 11397794 |
|  | 11432833 |
|  | 11448159 |
|  | 11477071 |
|  | 11551904 |
|  | 11641401 |
|  | 11689449 |
|  | 11704680 |
|  | 11708771 |
|  | 11739718 |
|  | 11801649 |
|  | 11823437 |
|  | 11867732 |
|  | 11877416 |
|  | 11891317 |
|  | 11903046 |
|  | 11912197 |
|  | 11914583 |
|  | 11948398 |
|  | 11956220 |
|  | 11983170 |
|  | 12052862 |
|  | 12077347 |
|  | 12080089 |
|  | 12087103 |
|  | 12091339 |
|  | 12165517 |
|  | 12167088 |
|  | 12183893 |
|  | 12186939 |
|  | 12437352 |
|  | 12446585 |
|  | 12485413 |
|  | 12547826 |
|  | 12554755 |
|  | 12592382 |
|  | 12600818 |
|  | 12615916 |
|  | 12618758 |
|  | 12682910 |
|  | 12692226 |
|  | 12700665 |
|  | 12799614 |
|  | 12805554 |
|  | 12853483 |
|  | 12875982 |
|  | 12909593 |
|  | 12949493 |
|  | 12954631 |
|  | 12963547 |
|  | 12963995 |
|  | 1310896 |
|  | 1448082 |
|  | 14500675 |
|  | 14511403 |
|  | 14522952 |
|  | 14530285 |
|  | 14532268 |
|  | 14645224 |
|  | 14652666 |
|  | 14654548 |
|  | 14667575 |
|  | 14739464 |
|  | 15001576 |
|  | 15034932 |
|  | 15087451 |
|  | 15158360 |
|  | 1516134 |
|  | 15191540 |
|  | 15226448 |
|  | 15249231 |
|  | 15262961 |
|  | 15271374 |
|  | 15302935 |
|  | 15314183 |
|  | 15314185 |
|  | 15333603 |
|  | 15351693 |
|  | 15378002 |
|  | 15383523 |
|  | 15385474 |
|  | 15469925 |
|  | 15480426 |
|  | 15507668 |
|  | 15514030 |
|  | 15528994 |
|  | 15530430 |
|  | 15544846 |
|  | 15550679 |
|  | 15579469 |
|  | 15586248 |
|  | 15642950 |
|  | 15654976 |
|  | 15701643 |
|  | 15702969 |
|  | 15718494 |
|  | 15746191 |
|  | 15863510 |
|  | 15870692 |
|  | 15894171 |
|  | 15897893 |
|  | 15922298 |
|  | 15923621 |
|  | 15923644 |
|  | 15936718 |
|  | 15941782 |
|  | 15991283 |
|  | 15994313 |
|  | 16023596 |
|  | 16055710 |
|  | 16140218 |
|  | 16158054 |
|  | 16199533 |
|  | 16223572 |
|  | 16236267 |
|  | 16243842 |
|  | 16253992 |
|  | 16285957 |
|  | 16289409 |
|  | 16303762 |
|  | 16328781 |
|  | 16341687 |
|  | 16343055 |
|  | 16361535 |
|  | 16380075 |
|  | 16410250 |
|  | 1651323 |
|  | 16569638 |
|  | 16571680 |
|  | 16582099 |
|  | 16620772 |
|  | 16690925 |
|  | 16702224 |
|  | 16786139 |
|  | 16844113 |
|  | 16887814 |
|  | 16895791 |
|  | 16905771 |
|  | 16911523 |
|  | 16928756 |
|  | 16980310 |
|  | 17085440 |
|  | 17097050 |
|  | 17101801 |
|  | 1827203 |
|  | 1922387 |
|  | 2138276 |
|  | 2320002 |
|  | 3194415 |
|  | 7662174 |
|  | 7666555 |
|  | 7690421 |
|  | 7738014 |
|  | 7816143 |
|  | 7823959 |
|  | 7848298 |
|  | 8087846 |
|  | 8108109 |
|  | 8137421 |
|  | 8302594 |
|  | 8380166 |
|  | 8387155 |
|  | 8397339 |
|  | 8440710 |
|  | 8464713 |
|  | 8557686 |
|  | 8570175 |
|  | 8617242 |
|  | 8628277 |
|  | 8649793 |
|  | 8654373 |
|  | 8663380 |
|  | 8663478 |
|  | 8733011 |
|  | 8763994 |
|  | 8798722 |
|  | 8837781 |
|  | 8974016 |
|  | 9129988 |
|  | 9154808 |
|  | 9211894 |
|  | 9223324 |
|  | 9235954 |
|  | 9334186 |
|  | 9346889 |
|  | 9405416 |
|  | 9445037 |
|  | 9468519 |
|  | 9488446 |
|  | 9510247 |
|  | 9545246 |
|  | 9566900 |
|  | 9621077 |
|  | 9628874 |
|  | 9642216 |
|  | 9670954 |
|  | 9671405 |
|  | 9685505 |
|  | 9732876 |
|  | 9786917 |
|  | 9872330 |
|  | 15984772 |
|  | 16033799 |
|  | 16799861 |
|  | 15489334 |
|  | 16586042 |
|  | 16676006 |
|  | 16710414 |
|  | 17009014 |
|  | 17121851 |
|  | 17130130 |
|  | 17215518 |
|  | 17235455 |
|  | 17237388 |
|  | 17237407 |
|  | 17283040 |
|  | 17283046 |
|  | 17314269 |
|  | 17317669 |
|  | 17401435 |
|  | 17403042 |
|  | 17466952 |
|  | 17506864 |
|  | 17510314 |
|  | 17588603 |
|  | 17634427 |
|  | 8662824 |
|  | 10582624 |
|  | 11980884 |
|  | 12376563 |
|  | 16916642 |
|  | 17042795 |
|  | 17145782 |
|  | 17182846 |
|  | 17189700 |
|  | 17244530 |
|  | 17270292 |
|  | 17308994 |
|  | 17341624 |
|  | 17428453 |
|  | 17430897 |
|  | 17442952 |
|  | 17468757 |
|  | 17500068 |
|  | 17554491 |
|  | 17596282 |
|  | 17699753 |
|  | 9447996 |
|  | 16874457 |
|  | 17216194 |
|  | 17503228 |
|  | 9651196 |
|  | 9847304 |

---

|  |  |
| --- | --- |
| CellType | hepatocyte |
|  | squamous cell |
|  | hematopoietic cell |
|  | transitional epithelial cell |
|  | smooth muscle myocyte |
|  | endometrial stromal cell |
|  | airway epithelial cell |
|  | decidual cell |
|  | macrophage |
|  | trophoblast |
|  | pneumocyte |
|  | germinal center B-cell |
|  | muscle cell |
|  | Purkinje cell |
|  | neuroglia |
|  | epidermis cell |
|  | Leydig cell |
|  | neuron |
|  | ovary follicle cell |
|  | kidney tubule cell |
|  | exocrine cell |
|  | islets of Langerhans |
|  | bile duct cells |
|  | glandular cells |
|  | Hepatocytes |
|  | squamous epithelial cells |
|  | lymphoid tissue |
|  | hematopoietic cells |
|  | urothelial cells |
|  | smooth muscle cells |
|  | cells in endometrial stroma |
|  | cells in red pulp |
|  | respiratory epithelial cells |
|  | cells in white pulp |
|  | decidual cells |
|  | Macrophages |
|  | cells in granular layer |
|  | trophoblastic cells |
|  | Pneumocytes |
|  | cells in molecular layer |
|  | germinal center cells |
|  | myocytes |
|  | Purkinje Cells |
|  | cells in seminiferus ducts |
|  | non-germinal center cells |
|  | glial cells |
|  | epidermal cells |
|  | Leydig Cells |
|  | neuronal cells |
|  | cells in glomeruli |
|  | follicle cells |
|  | cells in tubules |
|  | ovarian stroma cells |
|  | exocrine glandular cells |

---

|  |  |
| --- | --- |
| Tissue | smooth muscle |
|  | myocardium |
|  | mouth mucosa |
|  | skeletal muscles |
|  | lymphoid tissue |
|  | Pancreas |
|  | Liver |
|  | epididymis |
|  | esophagus |
|  | fallopian tube |
|  | Adrenal gland |
|  | Appendix |
|  | tonsil |
|  | bone marrow |
|  | urinary bladder |
|  | breast |
|  | uterus, post-menopause |
|  | spleen |
|  | parathyroid gland |
|  | bronchus |
|  | Placenta |
|  | Lung |
|  | Cerebellum |
|  | uterus, pre-menopause |
|  | stomach, lower |
|  | gall bladder |
|  | stomach, upper |
|  | Prostate |
|  | lymph node |
|  | heart muscle |
|  | vagina |
|  | testis |
|  | rectum |
|  | hippocampus |
|  | cerebral cortex |
|  | vulva/anal skin |
|  | Salivary gland |
|  | nasopharynx |
|  | thyroid gland |
|  | seminal vesicle |
|  | oral mucosa |
|  | Kidney |
|  | cervix, uterine |
|  | skeletal muscle |
|  | Ovary |
|  | Skin |
|  | lateral ventricle |
|  | colon |
|  | small intestine |
|  | duodenum |

---

|  |  |
| --- | --- |
| Organism | Homo sapiens {Organism urn:agi-taxid:9606} |
|  | Mus musculus {Organism urn:agi-taxid:10090} |
|  | Rattus norvegicus {Organism urn:agi-taxid:10116} |
|  | Homo sapiens |
|  | Mus musculus |
|  | Rattus norvegicus |
|  | Danio rerio |
|  | Bos taurus |
|  | Human |

---

|  |  |
| --- | --- |
| Mouse chromosome position | 4 43.34 cM |
|  | 4 44.6 cM |

---

|  |  |
| --- | --- |
| OMIM ID | 165160 |
|  | OMIM:165160 |

---

|  |  |
| --- | --- |
| Rat chromosome position | 5q33 |
|  | 5q34.1 |

---

|  |  |
| --- | --- |
| Hugo ID | 6204 |
|  | HGNC:6204 |

---

|  |  |
| --- | --- |
| Human chromosome position | 1p32.1 |
|  | 1p32-p31 |

---

|  |  |
| --- | --- |
| Swiss-Prot Accession | P05412 |
|  | P05412.2 |
|  | P05627 |
|  | Q52L79 |
|  | P05627.3 |
|  | P17325 |
|  | P17325.1 |
|  | Q6FHM7 |
|  | Q96G93 |
|  | Q6FHK0 |
|  | Q3US19 |
|  | Q3V255 |
|  | Q6TDG5 |
|  | JUN\_HUMAN |
|  | JUN\_MOUSE |
|  | JUN\_RAT |
|  | Q52L79\_MOUSE |

---

|  |  |
| --- | --- |
| PIR ID | A31264 |
|  | A31345 |
|  | S12742 |

---

|  |  |
| --- | --- |
| GenBank ID | NC\_000001 |
|  | NM\_002228 |
|  | NP\_002219 |
|  | NG\_047027 |
|  | AL136985 |
|  | AY217548 |
|  | AAO22993 |
|  | CH471059 |
|  | EAX06628 |
|  | CS542415 |
|  | CAM91157 |
|  | J04111 |
|  | AAA59197 |
|  | KT584072 |
|  | M23544 |
|  | U60581 |
|  | AK307137 |
|  | BC002646 |
|  | BC006175 |
|  | AAH06175 |
|  | BC009874 |
|  | AAH09874 |
|  | BC068522 |
|  | AAH68522 |
|  | BG831943 |
|  | BI793062 |
|  | BT019759 |
|  | AAV38564 |
|  | BU782554 |
|  | CD370800 |
|  | CR541724 |
|  | CAG46525 |
|  | CR541752 |
|  | CAG46552 |
|  | P05412 |
|  | NC\_000070 |
|  | NM\_010591 |
|  | NP\_034721 |
|  | AL732611 |
|  | CS693299 |
|  | CAP12263 |
|  | U03709 |
|  | AK132019 |
|  | BAE20944 |
|  | AK133722 |
|  | AK133867 |
|  | AK135023 |
|  | BAE22389 |
|  | AK140911 |
|  | BAE24518 |
|  | AK159196 |
|  | BAE34891 |
|  | AK159220 |
|  | AY423853 |
|  | AAR00344 |
|  | BC002081 |
|  | AAH02081 |
|  | BC021888 |
|  | AAH21888 |
|  | BC032086 |
|  | BC055887 |
|  | BC094032 |
|  | AAH94032 |
|  | BY106462 |
|  | J04115 |
|  | AAA37419 |
|  | X12740 |
|  | CAA31236 |
|  | X12761 |
|  | CAA31252 |
|  | P05627 |
|  | NC\_005104 |
|  | NM\_021835 |
|  | NP\_068607 |
|  | AC\_000073 |
|  | AABR07049287 |
|  | AAHX01038549 |
|  | CH473998 |
|  | EDL97766 |
|  | X17215 |
|  | CAA35084 |
|  | BC078738 |
|  | AAH78738 |
|  | BE113912 |
|  | BF414978 |
|  | CB740532 |
|  | CK475341 |
|  | X17163 |
|  | CAA35041 |
|  | P17325 |
|  | NC\_018912 |
|  | AMYH02001100 |
|  | DQ896432 |
|  | ABM87431 |
|  | AC\_000026 |
|  | AAHY01038209 |
|  | AC\_000133 |
|  | ABBA01074656 |
|  | AABR06038744 |
|  | CAC10201 |
|  | CAM14753 |
|  | NT\_032977 |
|  | NW\_001838579 |
|  | NW\_921351 |
|  | AC\_000044 |
|  | Q6FHK0 |
|  | Q6FHM7 |
|  | NT\_039280 |
|  | NW\_001030747 |
|  | Q3US19 |
|  | Q3V255 |
|  | Q52L79 |
|  | Q6TDG5 |
|  | NW\_001084842 |
|  | NW\_047717 |
|  | NW\_000210 |
|  | NW\_043856 |
|  | NT\_086582 |
|  | AF227190 |
|  | 51709812 |
|  | 6754401 |
|  | 6754402 |
|  | 12805238 |
|  | 12805239 |
|  | 21284396 |
|  | 21284397 |
|  | 21327814 |
|  | 192577 |
|  | 309169 |
|  | 52762 |
|  | 52763 |
|  | 52758 |
|  | 52759 |
|  | 135299 |
|  | 51458674 |
|  | 44890066 |
|  | 4758616 |
|  | 51460368 |
|  | 27805114 |
|  | 34870044 |
|  | 19923684 |
|  | 11177864 |
|  | 57079 |
|  | 57080 |
|  | 50927568 |
|  | 50927569 |
|  | 57819 |
|  | 57820 |
|  | 135300 |
|  | 27805115 |
|  | 186624 |
|  | 386839 |
|  | 38014305 |
|  | 13544091 |
|  | 13544092 |
|  | 40226266 |
|  | 40226267 |
|  | 46329589 |
|  | 46329590 |
|  | 14179530 |
|  | 15820787 |
|  | 54696383 |
|  | 54696384 |
|  | 23825888 |
|  | 31154890 |
|  | 49456408 |
|  | 49456409 |
|  | 49456462 |
|  | 49456463 |
|  | 135298 |
|  | 12314169 |
|  | AAF74120 |
|  | AF227191 |
|  | AAF74121 |
|  | 8248253 |
|  | 8248254 |
|  | 8248255 |
|  | 8248256 |
|  | AAH02646 |
|  | NT\_039263 |
|  | 123788411 |
|  | 123792303 |
|  | 37695626 |
|  | 62825871 |
|  | 74149179 |
|  | 74183908 |
|  | 74192749 |
|  | 74204894 |
|  | 81887398 |
|  | 81892901 |
|  | 119627033 |
|  | 123999845 |
|  | 74748524 |
|  | 74748528 |
|  | 33989472 |
|  | 41946923 |
|  | 55251229 |
|  | 62911209 |
|  | 82202287 |
|  | 82228700 |
|  | AAH56597 |
|  | AAH65976 |
|  | AAY21257 |
|  | BC056597 |
|  | BC065976 |
|  | CAH69118 |
|  | CR392368 |
|  | DQ003340 |
|  | NC\_007131 |
|  | NM\_199987 |
|  | NP\_956281 |
|  | NW\_001512107 |
|  | Q4ZJE9 |
|  | Q6NZT5 |
|  | 115304969 |
|  | 122065248 |
|  | 122132257 |
|  | 3212097 |
|  | AAC21576 |
|  | AAI23754 |
|  | AF069514 |
|  | BC123753 |
|  | NC\_007301 |
|  | NW\_001494814 |
|  | O77627 |
|  | Q08DH1 |
|  | XM\_001249361 |
|  | XP\_001249362 |
|  | 122890215 |
|  | 149044507 |
|  | XM\_216458 |
|  | XP\_216458 |
|  | BC056597,BC065976 |
|  | dre:335916 |
|  | 126007383 |
|  | 335916 |
|  | ZDB-GENE-030131-7859 |
|  | Dr.1064 |
|  | 41053400 |
|  | 41053401 |

---

|  |  |
| --- | --- |
| Swiss-Prot ID | JUN\_HUMAN |
|  | JUN\_MOUSE |
|  | JUN\_RAT |
|  | Q52L79\_MOUSE |

---

|  |  |
| --- | --- |
| Cell Localization | Nucleus |
|  | Cytoplasm |

---

|  |  |
| --- | --- |
| Ensembl ID | ENSG00000177606 |
|  | ENSP00000360266.2 |
|  | ENST00000371222.3 |
|  | ENSMUSG00000052684 |
|  | ENSMUSP00000102711.1 |
|  | ENSMUST00000107094.1 |
|  | ENSRNOG00000026293 |
|  | ENSRNOP00000011732.1 |
|  | ENSRNOT00000011731.3 |
|  | ENSP00000360266 |
|  | ENST00000371222 |
|  | ENSMUSP00000102711 |
|  | ENSMUST00000107094 |
|  | ENSRNOP00000011732 |
|  | ENSRNOT00000011731 |

---

|  |  |
| --- | --- |
| MGI ID | MGI:96646 |
|  | 96646 |

---

|  |  |
| --- | --- |
| RGD ID | 2943 |
|  | 69195 |

---

|  |  |
| --- | --- |
| Unigene ID | Hs.696684 |
|  | Mm.275071 |
|  | Rn.93714 |
|  | Rn.225373 |
|  | Hs.714791 |
|  | Hs.723911 |
|  | Hs.525704 |
|  | Rn.94975 |
|  | Hs.78465 |
|  | Rn.44320 |
|  | Mm.482 |
|  | Dr.1064 |
|  | Bt.11159 |

---

|  |  |
| --- | --- |
| Homologene ID | 1679 |

---

|  |  |
| --- | --- |
| Shape | O-vertex |

---

|  |  |
| --- | --- |
| IPI ID | IPI00121829 |
|  | IPI00215370 |
|  | IPI00008965 |

---

|  |  |
| --- | --- |
| Chromosome position | 1p32-p31 |
|  | 4 44.6 cM |
|  | 5q34.1 |

---

|  |  |
| --- | --- |
| Microarray ID | 1369788\_s\_at |
|  | A\_42\_P741027 |
|  | 100130\_at |
|  | 1417409\_at |
|  | 1448694\_at |
|  | msa.4586.0\_s\_at |
|  | x12761\_s\_at |
|  | A\_51\_P325914 |
|  | J04111 |
|  | 201464\_x\_at |
|  | 89913\_s\_at |
|  | 213281\_at |
|  | 201466\_s\_at |
|  | 201465\_s\_at |
|  | 32583\_at |
|  | 1895\_at |
|  | aa393106\_at |
|  | j04111\_at |
|  | rc\_d51060\_s\_at |
|  | A\_23\_P201538 |
|  | A\_14\_P127903 |
|  | A\_14\_P134030 |
|  | A\_14\_P109617 |
|  | M339 |
|  | R560 |
|  | OR0730 |
|  | 153 |
|  | OH7608 |
|  | MB488 |
|  | 1374404\_at |
|  | 1389528\_s\_at |
|  | rc\_AA944014\_at |
|  | rc\_AA945867\_at |
|  | rc\_AI175959\_at |
|  | X17163cds\_s\_at |
|  | 222125\_Rn |
|  | Msa.535.0\_at |
|  | A\_65\_P15588 |
|  | J0247B11-3 |
|  | NM\_010591 |
|  | 33353\_at |
|  | I\_931585 |

---

|  |  |
| --- | --- |
| Homo sapiens Chromosome position | 1p32-p31 |

---

|  |  |
| --- | --- |
| Ensembl Transcript ID | ENST00000371222 |

---

|  |  |
| --- | --- |
| ENSEMBL | ENSG00000177606 |

---

|  |  |
| --- | --- |
| FunctionalClass | DNA binding |
|  | RNA polymerase II transcription factor |
|  | double-stranded DNA binding |
|  | protein binding |
|  | protein heterodimerization |
|  | protein homodimerization |
|  | sequence-specific DNA binding |
|  | transcription factor |
|  | transcriptional activator |
|  | protein dimerization |
|  | transcription factor binding |
|  | transcription activator |

---

|  |  |
| --- | --- |
| Hugo Symbol | JUN |

---

|  |  |
| --- | --- |
| Bos taurus Chromosome position | 3 |

---

|  |  |
| --- | --- |
| Mus musculus Chromosome position | 4 44.6 cM |

---

|  |  |
| --- | --- |
| KEGG pathway | B cell receptor signaling pathway |
|  | Colorectal cancer |
|  | ErbB signaling pathway |
|  | Focal adhesion |
|  | GnRH signaling pathway |
|  | MAPK signaling pathway |
|  | Renal cell carcinoma |
|  | T cell receptor signaling pathway |
|  | Toll-like receptor signaling pathway |
|  | Wnt signaling pathway |

---

|  |  |
| --- | --- |
| State | p |

---

|  |  |
| --- | --- |
| Rattus norvegicus Chromosome position | 5q34.1 |

---

|  |  |
| --- | --- |
| Ensembl Gene ID | ENSG00000177606 |

---

|  |  |
| --- | --- |
| Keyword | transcription factor |
|  | nucleus |
|  | oncogene |
|  | golgi apparatus |
|  | bzip transcription factor |
|  | RAS small monomeric GTPase |
|  | leucine zipper |
|  | g protein coupled receptor |
|  | acetyltransferase |
|  | map kinase kinase |
|  | desmoplakin |
|  | liposome |
|  | DNA binding activity |
|  | binding activity |
|  | map kinases |
|  | basic leucine zipper |
|  | factor C |
|  | leucine repeat |
|  | desmoplakins |
|  | SAP kinase |
|  | DNA |
|  | RNA |
|  | nuclei |
|  | map kinase |
|  | activated blood coagulation factor XIV |
|  | JNK |

---

|  |  |
| --- | --- |
| ProteinAtlas NormalTissue | Tissue: lateral ventricle; Cell type: glial cells; Level: Low; Expression type: APE; Reliability: High |
|  | Tissue: appendix; Cell type: glandular cells; Level: Medium; Expression type: APE; Reliability: High |
|  | Tissue: duodenum; Cell type: glandular cells; Level: High; Expression type: APE; Reliability: High |
|  | Tissue: tonsil; Cell type: squamous epithelial cells; Level: None; Expression type: APE; Reliability: High |
|  | Tissue: pancreas; Cell type: exocrine glandular cells; Level: None; Expression type: APE; Reliability: High |
|  | Tissue: small intestine; Cell type: glandular cells; Level: Medium; Expression type: APE; Reliability: High |
|  | Tissue: kidney; Cell type: cells in tubules; Level: High; Expression type: APE; Reliability: High |
|  | Tissue: breast; Cell type: glandular cells; Level: Medium; Expression type: APE; Reliability: High |
|  | Tissue: colon; Cell type: glandular cells; Level: Medium; Expression type: APE; Reliability: High |
|  | Tissue: spleen; Cell type: cells in white pulp; Level: None; Expression type: APE; Reliability: High |
|  | Tissue: oral mucosa; Cell type: squamous epithelial cells; Level: High; Expression type: APE; Reliability: High |
|  | Tissue: skin; Cell type: epidermal cells; Level: Medium; Expression type: APE; Reliability: High |
|  | Tissue: adrenal gland; Cell type: glandular cells; Level: Low; Expression type: APE; Reliability: High |
|  | Tissue: appendix; Cell type: lymphoid tissue; Level: None; Expression type: APE; Reliability: High |
|  | Tissue: bone marrow; Cell type: hematopoietic cells; Level: None; Expression type: APE; Reliability: High |
|  | Tissue: bronchus; Cell type: respiratory epithelial cells; Level: Medium; Expression type: APE; Reliability: High |
|  | Tissue: cerebellum; Cell type: cells in granular layer; Level: Medium; Expression type: APE; Reliability: High |
|  | Tissue: cerebellum; Cell type: cells in molecular layer; Level: None; Expression type: APE; Reliability: High |
|  | Tissue: cerebellum; Cell type: Purkinje cells; Level: None; Expression type: APE; Reliability: High |
|  | Tissue: cerebral cortex; Cell type: glial cells; Level: Medium; Expression type: APE; Reliability: High |
|  | Tissue: cerebral cortex; Cell type: neuronal cells; Level: Low; Expression type: APE; Reliability: High |
|  | Tissue: cervix, uterine; Cell type: glandular cells; Level: None; Expression type: APE; Reliability: High |
|  | Tissue: cervix, uterine; Cell type: squamous epithelial cells; Level: Medium; Expression type: APE; Reliability: High |
|  | Tissue: epididymis; Cell type: glandular cells; Level: Low; Expression type: APE; Reliability: High |
|  | Tissue: esophagus; Cell type: squamous epithelial cells; Level: High; Expression type: APE; Reliability: High |
|  | Tissue: fallopian tube; Cell type: glandular cells; Level: Medium; Expression type: APE; Reliability: High |
|  | Tissue: gall bladder; Cell type: glandular cells; Level: High; Expression type: APE; Reliability: High |
|  | Tissue: heart muscle; Cell type: myocytes; Level: None; Expression type: APE; Reliability: High |
|  | Tissue: hippocampus; Cell type: glial cells; Level: None; Expression type: APE; Reliability: High |
|  | Tissue: hippocampus; Cell type: neuronal cells; Level: None; Expression type: APE; Reliability: High |
|  | Tissue: kidney; Cell type: cells in glomeruli; Level: None; Expression type: APE; Reliability: High |
|  | Tissue: lateral ventricle; Cell type: neuronal cells; Level: None; Expression type: APE; Reliability: High |
|  | Tissue: liver; Cell type: bile duct cells; Level: None; Expression type: APE; Reliability: High |
|  | Tissue: liver; Cell type: hepatocytes; Level: None; Expression type: APE; Reliability: High |
|  | Tissue: lung; Cell type: macrophages; Level: High; Expression type: APE; Reliability: High |
|  | Tissue: lung; Cell type: pneumocytes; Level: Medium; Expression type: APE; Reliability: High |
|  | Tissue: lymph node; Cell type: germinal center cells; Level: None; Expression type: APE; Reliability: High |
|  | Tissue: lymph node; Cell type: non-germinal center cells; Level: None; Expression type: APE; Reliability: High |
|  | Tissue: nasopharynx; Cell type: respiratory epithelial cells; Level: Medium; Expression type: APE; Reliability: High |
|  | Tissue: ovary; Cell type: ovarian stroma cells; Level: None; Expression type: APE; Reliability: High |
|  | Tissue: pancreas; Cell type: islets of Langerhans; Level: None; Expression type: APE; Reliability: High |
|  | Tissue: parathyroid gland; Cell type: glandular cells; Level: None; Expression type: APE; Reliability: High |
|  | Tissue: placenta; Cell type: decidual cells; Level: Medium; Expression type: APE; Reliability: High |
|  | Tissue: placenta; Cell type: trophoblastic cells; Level: None; Expression type: APE; Reliability: High |
|  | Tissue: prostate; Cell type: glandular cells; Level: None; Expression type: APE; Reliability: High |
|  | Tissue: rectum; Cell type: glandular cells; Level: Medium; Expression type: APE; Reliability: High |
|  | Tissue: salivary gland; Cell type: glandular cells; Level: Low; Expression type: APE; Reliability: High |
|  | Tissue: seminal vesicle; Cell type: glandular cells; Level: None; Expression type: APE; Reliability: High |
|  | Tissue: skeletal muscle; Cell type: myocytes; Level: Low; Expression type: APE; Reliability: High |
|  | Tissue: smooth muscle; Cell type: smooth muscle cells; Level: Low; Expression type: APE; Reliability: High |
|  | Tissue: spleen; Cell type: cells in red pulp; Level: None; Expression type: APE; Reliability: High |
|  | Tissue: stomach, lower; Cell type: glandular cells; Level: Medium; Expression type: APE; Reliability: High |
|  | Tissue: stomach, upper; Cell type: glandular cells; Level: Low; Expression type: APE; Reliability: High |
|  | Tissue: testis; Cell type: cells in seminiferus ducts; Level: None; Expression type: APE; Reliability: High |
|  | Tissue: testis; Cell type: Leydig cells; Level: None; Expression type: APE; Reliability: High |
|  | Tissue: thyroid gland; Cell type: glandular cells; Level: High; Expression type: APE; Reliability: High |
|  | Tissue: tonsil; Cell type: germinal center cells; Level: None; Expression type: APE; Reliability: High |
|  | Tissue: tonsil; Cell type: non-germinal center cells; Level: None; Expression type: APE; Reliability: High |
|  | Tissue: urinary bladder; Cell type: urothelial cells; Level: None; Expression type: APE; Reliability: High |
|  | Tissue: uterus, post-menopause; Cell type: cells in endometrial stroma; Level: Low; Expression type: APE; Reliability: High |
|  | Tissue: uterus, post-menopause; Cell type: glandular cells; Level: Low; Expression type: APE; Reliability: High |
|  | Tissue: uterus, pre-menopause; Cell type: cells in endometrial stroma; Level: Low; Expression type: APE; Reliability: High |
|  | Tissue: uterus, pre-menopause; Cell type: glandular cells; Level: None; Expression type: APE; Reliability: High |
|  | Tissue: vagina; Cell type: squamous epithelial cells; Level: High; Expression type: APE; Reliability: High |
|  | Tissue: vulva/anal skin; Cell type: epidermal cells; Level: High; Expression type: APE; Reliability: High |

---

|  |  |
| --- | --- |
| LocatorString | pmquery:curcumin prostate cancer |

---
